# Supplementary material for: The Mediating Role of Contextual Problems and Sensation Seeking in the Association between Substance Use and Mental Health in Adolescents from Northern Chile
Source: Int J Environ Res Public Health. 2022 Feb 17;19(4):2262. doi: 10.3390/ijerph19042262 (PMC8871919; doi:10.3390/ijerph19042262)
Supplement: Supplementary file 1 [file ijerph-19-02262-s001.zip › Complementary Material S1.pdf]

UNIVERSIDAD DE TARAPACÁ
ARICA - CHILE

CJE CENTRO JUSTICIA EDUCACIONAL

SET1- Preguntas demográficas

**Rut o documento de identidad del estudiante**

**Fecha**	

**Edad**

**Sexo**	
**Mujer**	
**Hombre**	

INSTRUCCIONES: MARCA CON UNA "X" LAS OPCIONES QUE CORRESPONDA.

Por favor, responde sinceramente. Tus respuestas contribuirán a servir de base para presentar propuestas de mejora al gobierno de Chile.

1. ¿En qué país naciste?

	Chile	
	Perú	
	Colombia	
	Venezuela	
	Bolivia	
	Ecuador	
	Argentina	
	Haití	
	Otro (especificar)	

2. ¿En qué país nacieron tus padres? (Favor contestar ambos)

**Mamá**	**Papá**		
		Chile	
		Perú	
		Colombia	
		Venezuela	
		Bolivia	
		Ecuador	
		Argentina	
		Haití	
		Otro (especificar)	

3. ¿Cuál es el nivel que cursas actualmente en el colegio?

1° básico			5° básico			1° medio	
2° básico			6° básico			2° medio	
3° básico			7° básico			3° medio	
4° básico			8° básico			4° medio	
